# Correction: Duburg et al. Composite Polybenzimidazole Membrane with High Capacity Retention for Vanadium Redox Flow Batteries. *Molecules* 2021, *26*, 1679

**DOI:** 10.3390/molecules27134234

**Published:** 2022-06-30

**Authors:** Jacobus C. Duburg, Kobra Azizi, Søren Primdahl, Hans Aage Hjuler, Elena Zanzola, Thomas J. Schmidt, Lorenz Gubler

**Affiliations:** 1 Electrochemistry Laboratory, Paul Scherrer Institut, CH-5232 Villigen, Switzerland; jacobus.duburg@psi.ch (J.C.D.); thomasjustus.schmidt@psi.ch (T.J.S.); lorenz.gubler@psi.ch (L.G.); 2 Blue World Technologies, Egeskovvej 6C, DK-3490 Kvistgård, Denmark; kaz@blue.world (K.A.); spr@blue.world (S.P.); hah@blue.world (H.A.H.); 3 Danish Center for Energy Storage, Frederiksholms Kanal 30, DK-1220 Copenhagen K, Denmark; 4 Laboratory for Physical Chemistry, ETH Zurich, CH-8093 Zurich, Switzerland

The authors wish to make the following changes to their paper [1].

## 2. Results

Original:

**Table 1:** V(IV) diffusion through NR212, FAP-450, and PP-PBI.

Name	Slope [V(IV)] vs. *t* (M∙L^−1^∙h^−1^)	V(IV) Diffusion (cm^2^∙min^−^^1^)
NR212	(650 ± 8) × 10^−6^	(744 ± 9) × 10^−8^
FAP-450	(259 ± 1) × 10^−6^	(351 ± 1) × 10^−8^
PP-PBI	(18 ± 2) × 10^−6^	(14 ± 1) × 10^−8^

To be replaced with:

**Table 1:** V(IV) diffusion through NR212, FAP-450, and PP-PBI.

Name	Slope [V(IV)] vs. *t* (M∙L^−1^∙h^−1^)	V(IV) Diffusion (cm^2^∙min^−^^1^)
NR212	(650 ± 8) × 10^−6^	(744 ± 9) × 10^−9^
FAP-450	(259 ± 1) × 10^−6^	(351 ± 1) × 10^−9^
PP-PBI	(18 ± 2) × 10^−6^	(14 ± 1) × 10^−9^

Explanation for the correction:

We observed an error in the calculations of the vanadium (IV) diffusion values; as a result of this, the order of magnitude of these values has been corrected to 10^−9^ cm^2^∙min^−1^ from 10^−8^ cm^2^∙min^−1^.

## 3. Discussion

Original:

V(IV) diffusion through the composite PP-PBI membrane was found to be the lowest ((14 ± 1) × 10^−8^ cm^2^∙min^−1^), while commercial Nafion^®^ NR212 suffered the highest V(IV) diffusion ((744 ± 9) × 10^−8^ cm^2^∙min^−1^),

To be replaced with:

V(IV) diffusion through the composite PP-PBI membrane was found to be the lowest ((14 ± 1) × 10^−9^ cm^2^∙min^−1^), while commercial Nafion^®^ NR212 suffered the highest V(IV) diffusion ((744 ± 9) × 10^−9^ cm^2^∙min^−1^).

Explanation for the correction:

We observed an error in the calculations of the vanadium (IV) diffusion values; as a result of this, the order of magnitude of these values has been corrected to 10^−9^ cm^2^∙min^−1^ from 10^−8^ cm^2^∙min^−1^.

## 4. Materials and Methods

Original:

The dry weight of the membrane (*w_dry_*) was obtained after drying it under vacuum at 55 °C for 22 h. The weight measurement was carried out in a closed vial to limit the uptake of moisture from the air. Then, the weight of the membrane in the wet state (*w_wet_*) was determined after immersion for 2 days in deionized water or in 1.6 M vanadium in 2 M H_2_SO_4_ and 0.05 M H_3_PO_4_ electrolyte (SOC −50%, 3.5 oxidation state, Oxkem, Reading, United Kingdom), followed by the removal of droplets on the surface with a tissue. In this case, the wet weight was measured in a vial to reduce the evaporation of water from the membrane. Lastly, water and electrolyte uptake of pristine *m*-PBI and of commercial membranes NR212 and FAP-450 was calculated according to Equation (1).

(1) $$\mathrm{Uptake}=\frac{w_{\mathrm{wet}}-w_{\mathrm{dry}}}{w_{\mathrm{dry}}}\cdot 100\%$$

To be replaced with:

The dry weight of the membrane (*m_dry_*) was obtained after drying it under vacuum at 55 °C for 22 h. The weight measurement was carried out in a closed vial to limit the uptake of moisture from the air. Then, the weight of the membrane in the wet state (*m_wet_*) was determined after immersion for 2 days in deionized water or in 1.6 M vanadium in 2 M H_2_SO_4_ and 0.05 M H_3_PO_4_ electrolyte (SOC −50%, 3.5 oxidation state, Oxkem, Reading, United Kingdom), followed by the removal of droplets on the surface with a tissue. In this case, the wet weight was measured in a vial to reduce the evaporation of water from the membrane. Lastly, water and electrolyte uptake of pristine *m*-PBI and of commercial membranes NR212 and FAP-450 was calculated according to Equation (1).

(1) $$\mathrm{Uptake}=\frac{m_{\mathrm{wet}}-m_{\mathrm{dry}}}{m_{\mathrm{dry}}}\cdot 100\%$$

Explanation for the correction:

The change described above has been made to be in line with the commonly used scientific unit of mass (*m*).

Original:

The measurements were carried out by filling two quartz cuvettes (Hellma Analytics, Zumikon, Switzerland) with 2.5 mL of solution from the MgSO_4_ flask. Each time, the measured solution was transferred back to the VOSO_4_ flask to avoid significant volume changes.

To be replaced with:

The measurements were carried out by filling two quartz cuvettes (Hellma Analytics, Zumikon, Switzerland) with 2.5 mL of solution from the MgSO_4_ flask. Each time, the measured solution was transferred back to the MgSO_4_ flask to avoid significant volume changes.

Explanation for the correction:

The correction described above has been made as the measured solutions were transferred back into the MgSO_4_ flask and not the VOSO_4_ flask.

Original:

Lastly, the change in weight (∆*w*) was calculated according to Equation (4). In Equation (4), *w_i_* and *w_f_* are the initial and final weight, respectively.

(4) $$\Delta w=\frac{wf-wi}{wi}\cdot 100\%$$

To be replaced with:

Lastly, the change in weight (∆*m*) was calculated according to Equation (4). In Equation (4), *m_i_* and *m_f_* are the initial and final weight, respectively.

(4) $$\Delta m=\frac{mf-mi}{mi}\cdot 100\%$$

Explanation for the correction:

The change described above has been made to be in line with the commonly used scientific unit of mass (*m*).

Original: 

Efficiencies and discharge capacity are calculated according to Equations (5)–(8). In Equations (5)–(7), *Q_ch_* and *Q_dis_* are the charges for the discharge and the charge process, while *V_dis_* and *V_ch_* are the discharge and charge volumes. In Equation (8), *Q_theoretical_* is the theoretical charge, *n* is the number of moles, F is the Faraday constant (96,485 C∙mol^−1^), and *z* is the charge.

(5) $$\eta C=\frac{Q_{\mathrm{dis}}}{Q_{\mathrm{ch}}}\cdot 100\%$$

(6) $$\eta V=\frac{V_{\mathrm{dis}}}{V_{\mathrm{ch}}}\cdot 100\%$$

(7) ηE=(ηC·ηV)·100%

(8) $$Q_{\mathrm{theoretical}}=I\cdot t=n\cdot (F\cdot z)$$

To be replaced with: 

Efficiencies and discharge capacity are calculated according to Equations (5)–(8). In Equations (5)–(7), *Q_ch_* and *Q_dis_* are the charges for the discharge and the charge process, while $\bar{U}$*_ch_* and $\bar{U}$*_dis_* are the average voltages during charge and discharge, respectively. In Equation (8), *Q_theoretical_* is the theoretical charge, *n* is the number of moles, F is the Faraday constant (96,485 C∙mol^−1^), and *z* is the number of electrons associated with the electrochemical reaction.

(5) $$\eta C=\frac{Q_{\mathrm{dis}}}{Q_{\mathrm{ch}}}\cdot 100\%$$

(6) $$\eta V=\frac{{\bar{U}}_{\mathrm{dis}}}{{\bar{U}}_{\mathrm{ch}}}\cdot 100\%$$

(7) ηE=(ηC·ηV)·100%

(8) $$Q_{\mathrm{theoretical}}=I\cdot t=n\cdot (F\cdot z)$$

Explanation for the correction:

The changes described above were made to avoid confusion between the average voltages in the cell ($\bar{U}$) and the unit volt (*V*). Furthermore, the description of this symbol was corrected to the average voltage instead of volume, which was a typing mistake. The last change was made to provide a clearer description of the symbol z as “charge” did not provide the desired clarity.

## 5. Conclusions

Original:

This asymmetric composite membrane showed the lowest V(IV) diffusivity ((14 ± 1) × 10^−8^ cm^2^∙min^−1^) as compared to the commercial Nafion^®^ NR212 and Fumasep^®^ FAP-450, (744 ± 9) × 10^−8^ and (351 ± 1) × 10^−8^ cm^2^∙min^−1^, respectively. 

To be replaced with:

This asymmetric composite membrane showed the lowest V(IV) diffusivity ((14 ± 1) × 10^−9^ cm^2^∙min^−1^) as compared to the commercial Nafion^®^ NR212 and Fumasep^®^ FAP-450, (744 ± 9) × 10^−9^ and (351 ± 1) × 10^−9^ cm^2^∙min^−1^, respectively. 

Explanation for the correction:

We observed an error in the calculations of the vanadium (IV) diffusion values; as a result of this, the order of magnitude of these values has been corrected to 10^−9^ cm^2^∙min^−1^ from 10^−8^ cm^2^∙min^−1^.

The authors apologize for any inconvenience caused and state that the scientific conclusions are unaffected. This correction was approved by the Academic Editor. The original publication has also been updated.
