# Supplementary material for: Composite Polybenzimidazole Membrane with High Capacity Retention for Vanadium Redox Flow Batteries
Source: Molecules. 2021 Mar 17;26(6):1679. doi: 10.3390/molecules26061679 (PMC8002762; doi:10.3390/molecules26061679)
Supplement: Supplementary file 1 [file molecules-26-01679-s001.zip › molecules-1119882-supplementary.pdf]

Supplementary Materials

Composite polybenzimidazole membrane with high capacity retention for vanadium redox flow batteries

Jacobus C. Duburg [1], Kobra Azizi [2], Søren Primdahl [2], Hans Aage Hjuler [2,3], Elena Zanzola [1,*], Thomas J. Schmidt [1,4] and Lorenz Gubler [1]

1 Electrochemistry, Laboratory, Paul Scherrer Institut, CH-5232 Villigen, Switzerland; jacobus.duburg@psi.ch (J.C.D.); thomasjustus.schmidt@psi.ch (T.J.S.); lorenz.gubler@psi.ch (L.G.)
2 Blue World Technologies, Egeskovvej 6C, DK-3490 Kvistgård, Denmark; kaz@blue.world (K.A.); spr@blue.world (S.P.); hah@blue.world (H.A.H.)
3 Danish Center for Energy Storage, Frederiksholms Kanal 30, DK-1220 Copenhagen K, Denmark
4 Laboratory for Physical Chemistry, ETH Zurich, CH-8093 Zurich, Switzerland
* Correspondence: elena.zanzola@psi.ch; Tel.: +41 56 310 4738

Contents:

Figure S1. Proton nuclear magnetic resonance (^{1}H NMR) spectrum of *m*-PBI.
Figure S2. Thermo-gravimetric analysis and differential thermal analysis (TGA/DTA) of *m*-PBI film.
Figure S3. Fourier transform spectroscopy (FTIR) of the pristine and acid doped *m*-PBI film.
Table S1. Water and electrolyte uptake, in-plane conductivity and accelerated stress test of NR212, FAP-450 and *m*-PBI.
Figure S4. Calibration plot of $VOSO_4$.
Figure S5. Photo of the "home-built" diffusion cell for testing the V(IV) diffusion through the membranes (NR212, FAP-450 and PP-PBI).
Figure S6. EDX map of the PP-PBI membrane after 90 consecutive galvanostatic cycles in the VRFB at 120 mA·cm^{-2}.
Figure S7. Cell components of the VRFB setup used in the present work.
Figure S8. Ex situ stability test of *m*-PBI in V(V) electrolyte.

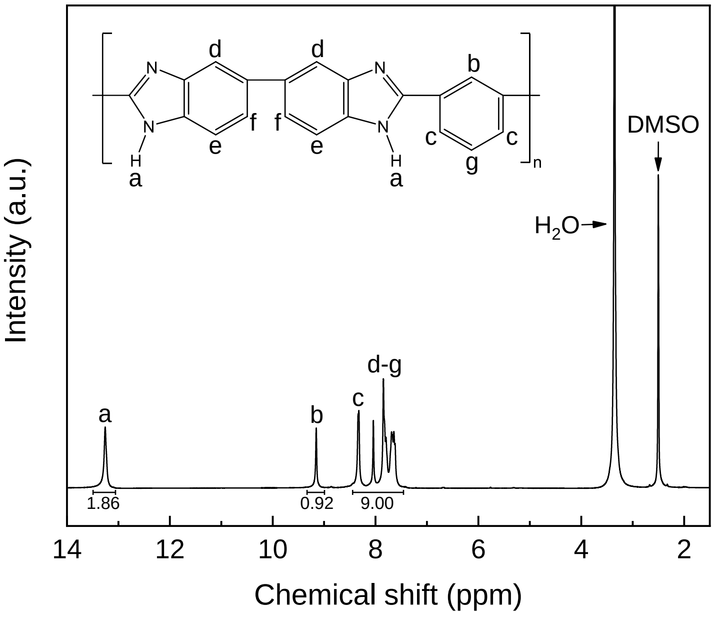

Figure S1. ^{1}H NMR spectrum of *m*-PBI powder in DMSO-d_6.

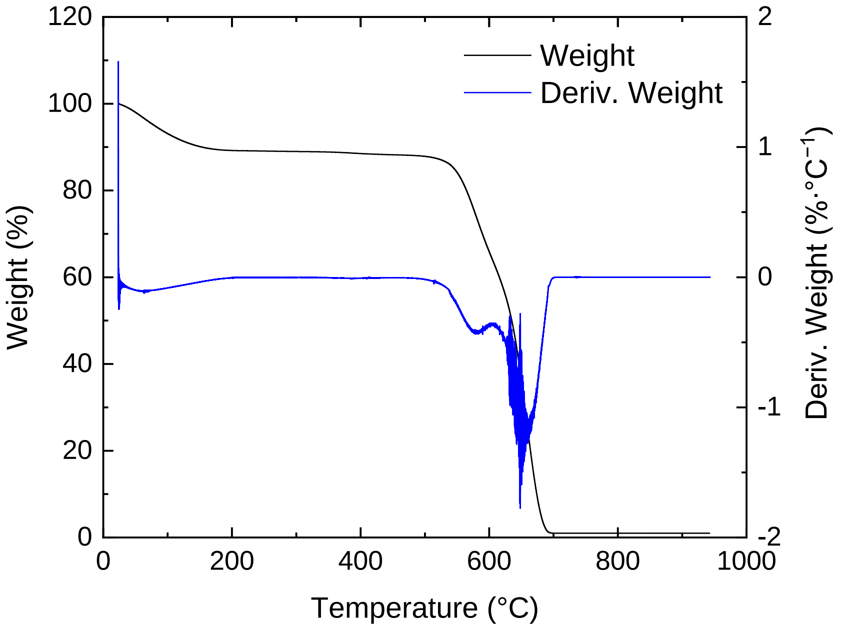

Figure S2. TGA/DTA analysis on the *m*-PBI film.

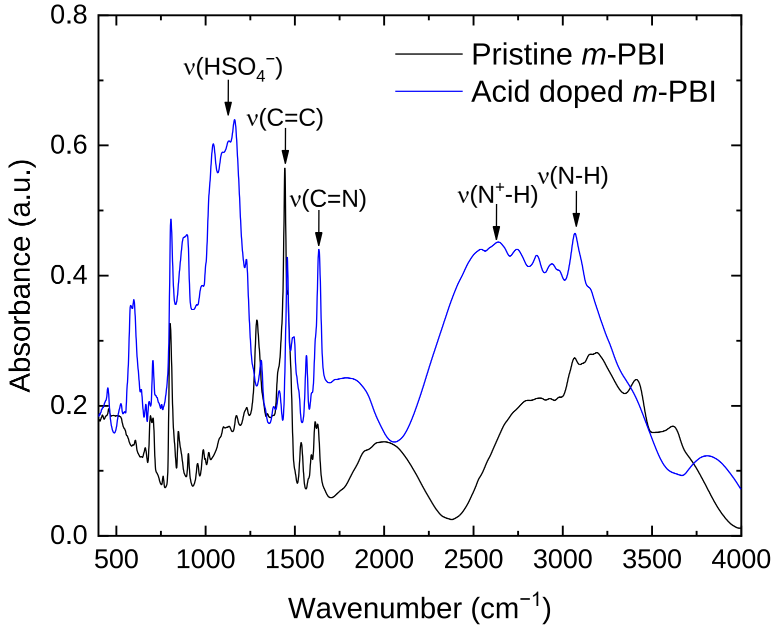

Figure S3. FTIR of the pristine and the acid doped *m*-PBI film. The characteristic peaks were assigned according to [1-3].

Table S1. Results of the water and electrolyte uptake, in-plane conductivity and accelerated stress test of the NR212, FAP-450 and *m*-PBI membranes.

Name	Thickness Dry state (µm)	Thickness Wet state (µm)	Water uptake (wt. %)	Electrolyte uptake (wt. %)	In-plane conductivity ($mS \cdot cm^{-1}$)	Accelerated stress test Weight change (%)
NR212	50.5 ± 0.3	51.8 ± 0.6	17.9 ± 1.5	6.8 ± 0.7	19.4 ± 0.2	-2.6 ± 0.3
FAP-450	55.2 ± 2.5	61.2 ± 2.5	7.4 ± 0.8	45.9 ± 1.0	N/A*	1.3 ± 0.2
m-PBI	38.8 ± 1.0	46.8 ± 0.6	15.8 ± 1.3	74.7 ± 0.6	0.74 ± 0.18	-74.2 ± 9.6**

*The in-plane conductivity of FAP-450 was too unstable to be recorded.

**Despite the fact that the *m*-PBI showed a good oxidative resistance in the ex situ stability test performed in V(V) (indeed, according to information from the industry, source undisclosed, PBI materials show high stability in high purity vanadium electrolyte, suitable for application in the field), the accelerated stress test (AST) displayed a significant degradation. As the AST had not been implemented by Oldenburg *et al*. [4] using PBI based membranes but amphoteric ion exchange membranes, it is unsure if it is applicable to the *m*-PBI membrane. The correlation between the measured degradation of the *m*-PBI membrane in the AST and in the ex situ test in V(V) needs to be studied in the future for a better understanding of its applicability to *m*-PBI based membranes.

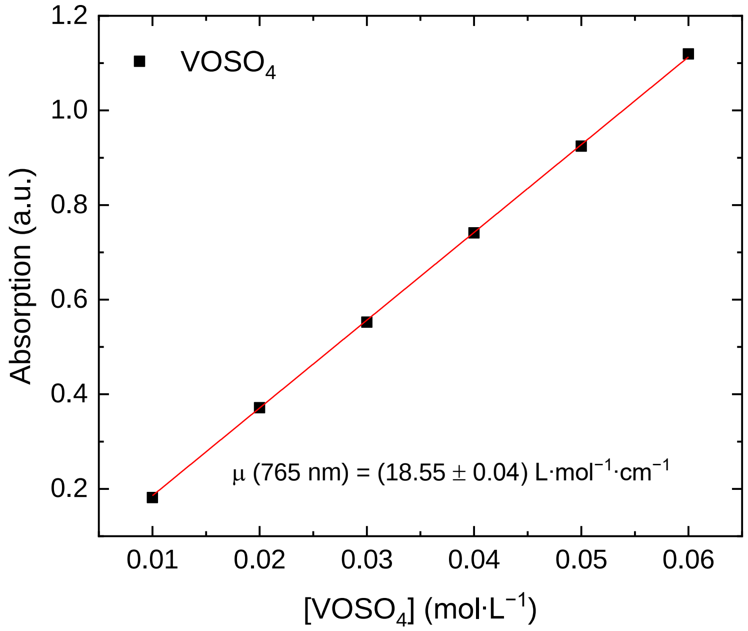

Figure S4. Calibration plot of $VOSO_4$ in 2 M H_2SO_4.

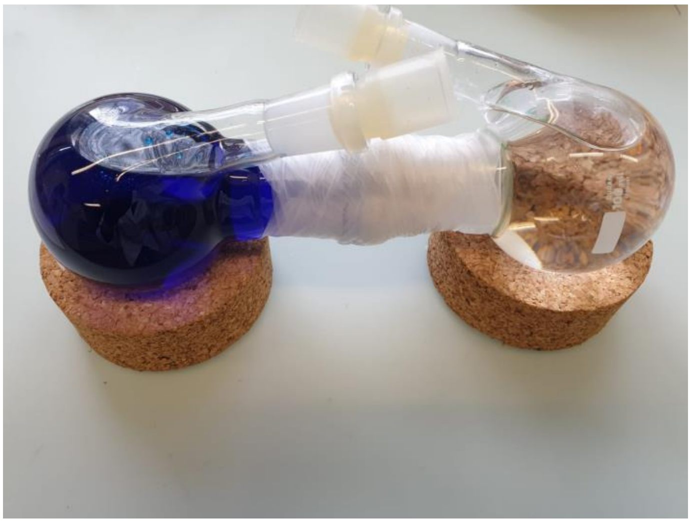

Figure S5. Photo of the "home-built" diffusion cell for testing the V(IV) diffusion through the membranes (NR212, FAP-450 and PP-PBI).

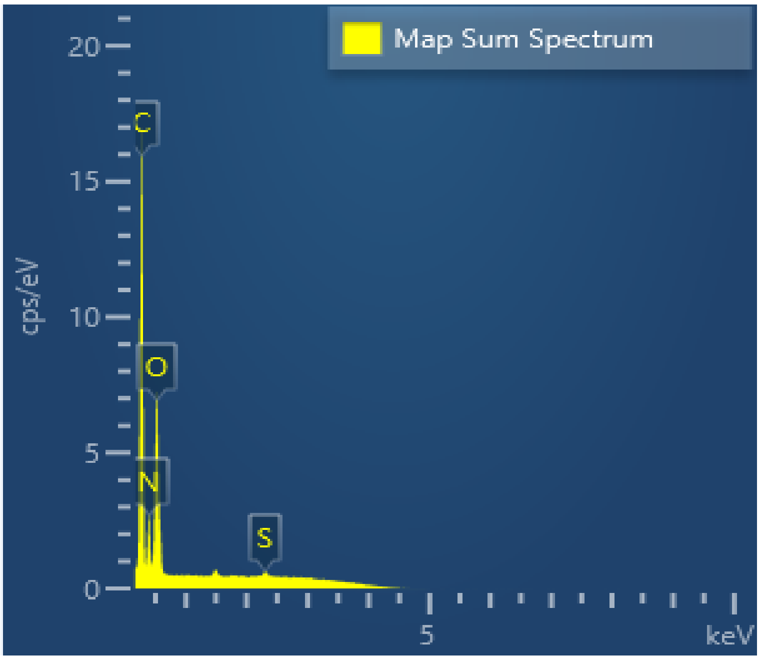

Figure S6. EDX map of the PP-PBI membrane after 90 consecutive galvanostatic cycles in the VRFB at 120 mA·cm^{-2}.

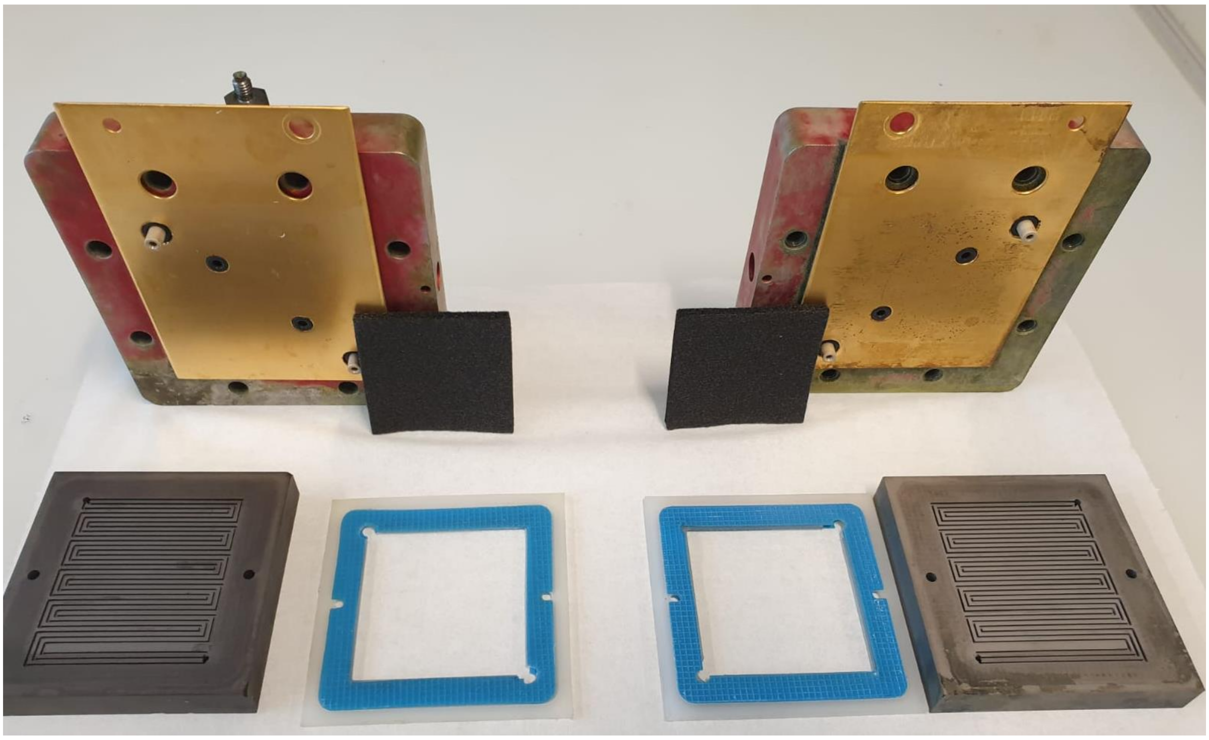

Figure S7. Cell components of the VRFB setup used in the present work.

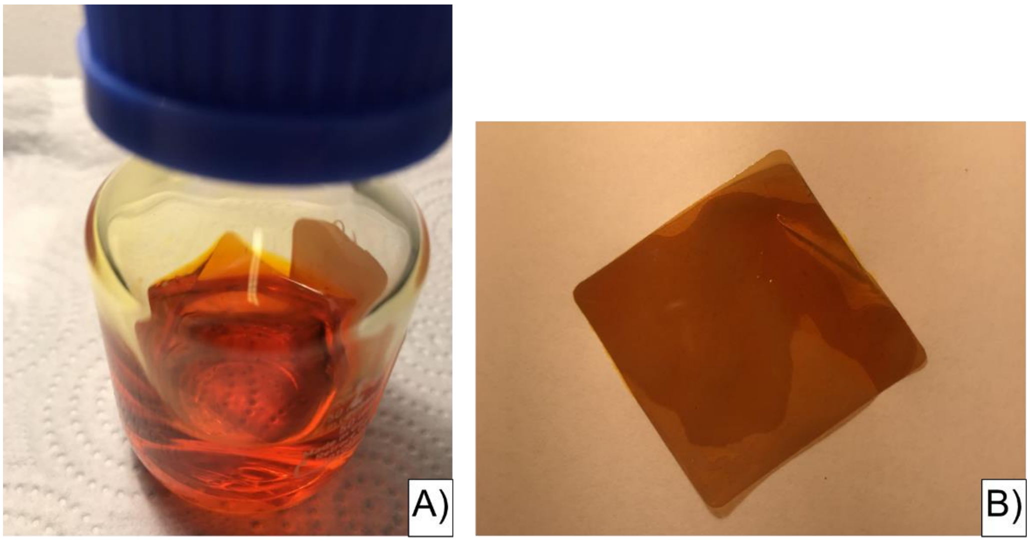

Figure S8. Photos of the ex situ stability test of the *m*-PBI membrane in V(V) electrolyte.

References

1. Bouchet, R.; Siebert, E., Proton conduction in acid doped polybenzimidazole. *Solid State Ionics* **1999,** *118*, 287–299.
2. Giffin, G. A.; Conti, F.; Lavina, S.; Majerus, A.; Pace, G.; Korte, C.; Lehnert, W.; Di Noto, V., A vibrational spectroscopic and modeling study of poly(2,5-benzimidazole) (ABPBI) – Phosphoric acid interactions in high temperature PEFC membranes. *International Journal of Hydrogen Energy* **2014,** *39*, 2776–2784.
3. Aldrich, S. IR spectrum table & chart. Available online: https://www.sigmaaldrich.com/technical-documents/articles/biology/ir-spectrum-table.html (accessed on 27 January 2020).
4. Oldenburg, F. J.; Ouarga, A.; Schmidt, T. J.; Gubler, L., Accelerated Stress Test Method for the Assessment of Membrane Lifetime in Vanadium Redox Flow Batteries. *ACS Applied Materials & Interfaces* **2019,** 11, 47917–47928.
